# Person-Centered Practice in the Portuguese Healthcare Services: A Scoping Review Protocol

**DOI:** 10.3390/nursrep12010024

**Published:** 2022-03-18

**Authors:** Filipa Ventura, Cristina R. B. Costeira, Rosa Silva, Daniela Cardoso, Cláudia Oliveira

**Affiliations:** 1 The Health Sciences Research Unit: Nursing (UICISA: E), Nursing School of Coimbra (ESEnfC), 3000 Coimbra, Portugal; cristina.costeira@ipleiria.pt (C.R.B.C.); rosacgsilva@esenfc.pt (R.S.); dcardoso@esenfc.pt (D.C.); cjoliveira@ualg.pt (C.O.); 2 Center for Innovative Care and Health Technology (ciTechcare), Polytechnic of Leiria, 2411 Leiria, Portugal; 3 School of Health Sciences, Polytechnic of Leiria, 2411 Leiria, Portugal; 4 Portugal Centre for Evidence Based Practice: A JBI Centre of Excellence (PCEBP/JBI), 3000 Coimbra, Portugal; 5 School of Health Jean Piaget Algarve, Piaget Institute, 8300 Silves, Portugal

**Keywords:** ethics, healthcare delivery, narrative, partnership, patient-centered care, person-centeredness, person-centered integrated care, Portugal

## Abstract

Recognizing the importance of the international advancements on person-centered practice (PCP) with positive implementation outcomes at the varied levels of healthcare systems, this scoping review will examine the PCP in Portuguese healthcare services. The Joanna Briggs Institute (JBI) guidance for scoping reviews will be followed. The Population (P) Concept (C) Context (C) mnemonic will scaffold research questions, the inclusion and exclusion criteria, and the searching strategy. Literature reporting on person-centeredness domains at the macro-, meso-, and micro levels applied to Portuguese healthcare services in Portuguese and English will be considered for inclusion. Accordingly, MEDLINE, CINAHL, SCOPUS, LILACS, SCIELO, Open Access Scientific Repository of Portugal (RCAAP), and Open gray will be searched. The literature will be screened for eligibility by two independent reviewers, first by title and abstract and subsequently by full text. A data extraction matrix designed to answer the research questions will be used for the included literature. The charted data will be thematically analyzed and presented graphically, with a narrative description of the literature characteristics. The results are expected to inform healthcare stakeholders at varying levels about the PCP domains where further improvements might be required in order to raise the quality of care to the international gold standards.

## 1. Introduction

Person-centered practice (PCP) has become commonplace in contemporary society as an essential cornerstone of sustainable healthcare services [1]. In 2015, the World Health Organization (WHO) reinforced the importance of changing the paradigm of care provision to person-centered models that integrate the perspectives of individuals, families, communities, both as participants in the co-development of services, and as users, according to their expectations, preferences, and needs in a humane and holistic way. The WHO global strategy on people-centered and integrated health services further recognized the importance of a work environment that allows the care provider to achieve full functioning [2].

In Europe, the United Kingdom and the Scandinavian countries are contributing with sustainable approaches to the systematic and coherent development, implementation, and evaluation of the PCP. In this context, two research centers are pioneers in the development of theoretical references for the practice, research, and teaching of PCP. The Queen Margaret University, Edinburg, Scotland, hosts the Center for Person-centered Practice Research (CPcPR). Underpinned by the Human Flourishing ethics and processes of critical creativity, person-centered practice is defined as an approach to practice established through the development and promotion of healthful relationships among healthcare staff, users of healthcare services, and others who are significant to them [3]. At the core of person-centeredness, McCormack [4] emphasizes interpersonal relationships as a means of being, becoming, and transforming. In that context, the narrative is seen as a holistic way of understanding the meaning a person gives to the world. Additionally, context is crucial to shape the person’s experience and generate emotions [4].

These principles of personhood and person-centeredness impregnate the person-centered practice framework (PCPF), which circularly depicts the complexity associated with the articulation of five essential domains toward the promotion of person-centeredness of healthcare practice: the macro context, the prerequisites, the practice environment, the person-centered processes and the outcomes [5].

The University of Gothenburg Center for Person-Centered Care (GPCC) posits person-centered care as applied ethics to healthcare. Derived from the ethics of Paul Ricœur, the GPCC model is underpinned by the ethical standpoint of “living the good life, with and for others in just institutions” and comprises three routines to its’ implementation: the narrative, the partnership, and the documentation of partnership in a health plan [6].

The GPCC model has been particularly influential in the development of the European standard EN 17398:2020 for patient involvement in health care, establishing the minimum requirements for person-centered care. The standard development was a multinational effort undertaken by the CEN/TC 450 Technical Committee starting from the central dimensions of the GPCC model CCP (i.e., narrative, partnership and documentation, and information sharing) and is the first to be published in the PCP domain [7]. The EN 17398:2020 is intended to facilitate the work of varied stakeholders in the introduction, development, and research on PCP across health services, users representatives, researchers, or enterprises. It might also be useful to managers and administrators, as well as actors in the political system, in the sense of informing metaprograms with the dimensions necessary for PCP [7].

Concerning the domain of implementation of PCP, the GPCC led a multinational collaboration (i.e., COST-Cares project), wherein the we-care roadmap was developed to assist the implementation of PCP across healthcare systems aiming at their sustainability. The roadmap starting point depicts person-centered care and health promotion as essential driving forces of accessible, high-quality, and sustainable healthcare services. Additionally, five critical enablers of implementation of PCP are identified: technology, quality strategies, infrastructure, incentive systems, and hiring strategies. In a viability study, the roadmap allowed the transferability of the GPCC model of person-centered care, leading to reduced healthcare costs without harming the quality of care [8].

In the implementation domain of PCP, the conceptual map to the practice of person-centered care is an additional important reference. Departing from a narrative review, the conceptual maps synthesized the evidence on person-centered care according to the Donabedian model of quality of care [9]. The structural domain is related to the healthcare system or the specific healthcare delivery context and is considered the essential pillar to (a) create a culture of PCP transversal to the care process, (b) co-design of education programs, as well as health promotion and disease prevention programs with healthcare service users, (c) establish a supportive environment, (d) develop and integrate supportive structures for information and communication technologies in health, (e) evaluate and monitor the implementation of the PCP. The procedural domain is associated with the interaction between healthcare providers and users, a description of the importance of (a) cultivating communication and healthcare processes with dignity and compassion, (b) involving the user in the management of their health/illness processes, and (c) integrated care. Concerning the outcomes, access to healthcare and self-reported outcomes are identified to reflect the implementation of PCP from the interaction between the healthcare system, healthcare providers, and service users [9].

Altogether, the theoretical frameworks mapping person-centeredness domains of PCP and its’ implementation have brought consistency to the international discourse and prompted the development of policy, standards, and evidence on the positive outcomes at the macro, meso, and micro levels of healthcare systems [6,8,10]. Even though PCP is reflected more or less extent in the discourse of both healthcare professionals, educators, and researchers, as well as service users, the developments in its’ implementation do not occur at equal rhythms across societies [11]. A recent integrative review of the evidence on person-centered care denotes that the concept is still ill-defined, which might hamper its assessment and implementation in clinical settings [12].

Particularly in Portugal, the National Health Service (SNS) follows the European agenda in the shift toward person-centered healthcare. The ‘SNS + Proximity’ is recognized as the kick-off effort to meet the needs and expectations of citizens, recognizing the central role of the person in the management of their health/illness processes and in the co-development of healthcare services [13]. Another element pointing toward the value of the person in Portuguese healthcare organizations is reflected in the National Health Assessment System (SINAS), where the person-centeredness of care is one of the five dimensions in the assessment of the quality of care provided [14]. At the policy level, the dispatch no. 9323-A/2018 Office of the Secretary of State for Health published 3 October 2018 in the Diário da República, 2nd series, no. 191, refers to the importance of improving the quality of services through a change in the paradigm of care provision, which should be reorganized around the citizen, focusing on their needs and expectations [15].

Recognizing the importance of the international advancements in the field to understand the state-of-the-art of the Portuguese PCP at the macro, meso, and micro levels will allow identifying where further improvements are required in order to enhance the quality of care. A preliminary search of PROSPERO, MEDLINE, and LILACS was conducted, and no ongoing scoping reviews on the person-centered practice in Portuguese healthcare services were identified.

Accordingly, the proposed scoping review will aim to examine and map PCP at the macro-, meso-, and micro levels in the Portuguese healthcare services. The identified evidence will be specifically characterized by addressing the following specific questions:

- What is the Portuguese legislation related to person-centered practice?
- What are the regulatory standards guiding PCP in Portuguese healthcare settings?
- What are the best practice guidelines related to PCP in Portuguese healthcare settings?
- What are the philosophical underpinnings of PCP in Portuguese healthcare settings?
- What are the processes of PCP being applied in Portuguese healthcare settings?
- What are the effectiveness and process outcomes of PCP implementation in Portuguese healthcare settings?
- What are the instrument measures being used to assess the effectiveness and process outcomes of PCP implementation in Portuguese healthcare settings?

## 2. Methods

The proposed literature review will adhere to the PRISMA-ScR guidelines and in accordance with the JBI methodology for scoping reviews [16]. Accordingly, the Population (P) Concept (C) Context (C) mnemonic will scaffold the inclusion and exclusion criteria and the design of the searching strategy. The protocol has been registered at OSF (DOI: 10.17605/OSF.IO/N3YKQ).

### 2.1. Inclusion Criteria

#### 2.1.1. Population

The proposed review will consider literature that reports on PCP carried out by healthcare professionals to healthcare service users in Portuguese healthcare care settings. Healthcare professionals entail, but are not limited to, nurses, physicians, psychologists, physiotherapists, nutritionists, or midwives. Literature involving non-healthcare professionals will be excluded. Healthcare service users include all persons participating in healthcare processes with healthcare professionals (i.e., seeking or receiving healthcare), which might include but are not limited to persons of all ages, patients of all ages, relatives, family, community groups, residents at assisted living facilities.

#### 2.1.2. Concept

In the absence of a unified definition of PCP [12], the Donabedian model will be followed to scaffold the elements of structure, process, and outcomes [17]. Accordingly, the proposed review will consider literature that reports on PCP that might be defined as a healthcare intervention or approach to providing healthcare that addresses the following structural domains [5,6,9]:

- At the macro level: policy or standards related to PCP;
- At the meso level: the organizational context is guided by person-centeredness principles underpinned on an ethical standpoint that guides the healthcare professionals’ actions as fellow human beings toward healthcare service users;
- At the micro level: the care environment enables PCP through, but not limited to, the promotion of healthful relationships and ambition for high quality of care;
- At the micro level: PCP is anchored on evidence-based guidelines.

Concerning the processes through which PCP occurs, it might entail routines [6] such as:

1. The narrative of the healthcare service user;
2. The establishment of a therapeutic partnership between the service user, their relatives, and healthcare professionals;
3. The existence of a health plan.

Other processes of PCP that will similarly be considered for inclusion are working with the person’s beliefs and values, shared decision making, authentic engagement, sympathetic presence, and working holistically [5].

Studies reporting on effectiveness and process outcomes of PCP implementation will be considered for inclusion when pertaining to person-centered outcomes (e.g., self-efficacy), patient-reported outcomes (e.g., distress, fatigue, pain, wellbeing), or process outcomes (e.g., accessibility to care, cost, quality of care, satisfaction) [5,9,18].

#### 2.1.3. Context

The prospective review will consider literature reporting on PCP at all Portuguese healthcare settings irrespective of their healthcare level (i.e., primary, secondary, or tertiary), including community healthcare or geographical location (i.e., urban, rural). Studies pertaining to non-Portuguese healthcare settings will be excluded.

#### 2.1.4. Types of Sources

In this scoping review, studies following quantitative, qualitative, and mixed methods designs will be considered for inclusion. Furthermore, legislation, healthcare standards, guidelines for evidence-based practice, and text and opinion papers will be considered for inclusion in the proposed scoping review.

### 2.2. Search Strategy

The search strategy will aim to locate both published and unpublished primary studies, legislation, healthcare standards, guidelines for evidence-based practice, and text and opinion papers published in Portuguese and English. An initial limited search of MEDLINE (PubMed) and CINAHL (EBSCO) was undertaken to identify articles on the topic. The text words contained in the titles and abstracts of relevant articles and the index terms used to describe the articles were used to develop a full search strategy for MEDLINE (PubMed), CINAHL (EBSCO), SCOPUS, LILACS, and SCIELO (Table A1, Appendix A). Sources of unpublished studies and gray literature to be searched include Open Access Scientific Repository of Portugal (RCAAP) and Open gray. As each database has its specificities concerning the way by which the information is organized, the search strategy, along with the keywords and index terms, will be adapted for each of the selected databases. The reference lists of articles included in the review will be screened for additional papers matching the inclusion and exclusion criteria. Guidelines related to evidence-based practice will be retrieved from the Directorate-General of Health of Portugal (DGS). Governmental policy related to health will be looked for at the Regulatory Authority of Health (ERS) and Diário da República.

This stage will be initiated upon the article’s acceptance for publication and is expected to be finished within one month.

### 2.3. Study/Source of Evidence Selection

Following the search, all identified records will be collated and uploaded into EndNote 20 (Clarivate Analytics, Philadelphia, PA, USA) will be used to assist in uploading, listing, and duplicating removal of the records identified through the search strategy. The criteria for study selection will be pilot tested within the research team. Sequentially, titles and abstracts will be screened by two independent researchers against the review’s inclusion/exclusion criteria. Prospective articles will be retrieved in full and imported into the Rayyan. The full text of selected citations will then be assessed in detail against the inclusion criteria by two independent reviewers. Reasons for the exclusion of full-text papers will be recorded and reported in the scoping review. Any disagreements arising between the reviewers at each stage of the selection process will be resolved through discussion or with a third reviewer. The results of the search will be reported in full in the final scoping review and presented in a PRISMA-ScR flow diagram [19].

This stage is expected to extend for one month but might be longer depending on the total number of retrieved records from all databases.

### 2.4. Data Extraction

Two independent reviewers will extract data from papers selected for inclusion by following the data extraction matrix (cf. Table A2 see Appendix B). The data extracted will include specific details about the healthcare professionals and users of healthcare services, PCP structure, process and outcomes, healthcare context, type of literature, study design when appropriate, and other findings relevant to the review question. The data will be exported to Microsoft Excel (Redmond, Washington, DC, USA). The preliminary data extraction matrix developed by the reviewers will be changed and revised as necessary during data extraction. Eventual modifications will be reported in detail in the scoping review report. Any disagreements that arise between the reviewers will be resolved through discussion or with a third reviewer. Where required, the authors of the articles or documents will be contacted by the research team to request additional data.

This stage is expected to take at least two months in light of the already count of unscreened records.

### 2.5. Data Analysis and Presentation

The charted data will be synthesized and thematically analyzed [20] following the data extraction domains relating to person-centeredness elements of structure, process, and outcomes of PCP in healthcare contexts with a narrative description of the literature characteristics. From this analysis, a secondary synthesis of the evidence on PCP at the macro, meso, and micro levels will be provided.

Tables and diagrams followed by a narrative summary will be used to present the results so that they sequentially address the scoping review-specific research questions. This stage will include the final scientific research report and is expected to take one month. Accordingly, with the previous stages initiated in March 2022 and occurring isolated and sequentially, this scoping review is expected to be concluded by the beginning of August 2022.

## 3. Discussion

In light of the international evidence, elements of person-centeredness need to be considered at several levels in order to promote PCP and person-centered outcomes [5,8]. These levels might entail the macro context pertaining to policy and regulation, the meso context entailing organizational leadership culture and communication structure, from which the interplay influences the micro-context [8]. This scoping review is designed to particularly address these varied levels, namely with regards to national and international strategic approaches and health and social care policies, as it will include regulatory documents searched at the Portuguese Health Authorities websites.

At the micro-context where the encounter between the person and the healthcare staff occurs, the PCP framework depicts the domains of prerequisites, care environment characteristics, and processes of care that, if in place, allow for accomplishing person-centered outcomes [8]. The data extraction matrix was designed to capture the sub-constructs in the above-mentioned domains.

Briefly, the prerequisites identify the attributes of healthcare professionals that reflect a person-centered professional with the ability to adapt to the challenges of a changing context. These prerequisites include being professionally competent, with developed interpersonal skills, self-knowledge, clarity of beliefs and values, and commitment to work. However, each healthcare professional and the team as a whole express and practice these attributes, the final outcome of PCP will not be achieved if the care environment is not a facilitator of it.

The domain pertaining to the care environment represents the healthcare context, and its complexity has received particular attention along with the development of implementation science. Specifically, within the scope of the PCP, seven elements that promote person-centeredness are considered: organizational support systems, potential for innovation and risk-taking, physical environment, power sharing, effective interprofessional relationships, shared decision-making systems, and diversity of skills.

Finally, person-centered processes constitute the operationalization of the CFP and include working with the person’s beliefs and values, shared decision making, authentic involvement, having an empathetic presence, and working holistically. This domain specifically focuses on the relationship with the person-user in the context of care provision. Each of the domains and their articulation at various levels will be necessary to achieve the final outcome of a person-centered practice, i.e., a healthful culture in healthcare perceived by both users and employees. Expected results of a person-centered practice might therefore be good care experiences, involvement in care, and feeling of wellbeing [5].

To the PCP framework elements of person-centeredness, the GPCC model further adds three activities that allow practicing person-centered care, i.e., the narrative, the partnership, and the health plan [6]. The GPCC activities were usefully integrated into the domain of person-centered processes for the purpose of data extraction. However, the authors are aware that such activities demand communicational and relational prerequisites from the professional to enable seeing the person-patient as a unique human being with fragilities as well as resources [6].

Importantly, all these elements must be thought of from a multiprofessional perspective. The inclusion of the extraction fields to characterize the population and the context will allow identifying the extent to which the different professional domains attend to PCP and at which levels of care (e.g., primary care, community care, elderly care, specialized care).

In light of the results from the test to the preliminary searching strategy on MEDLINE (PubMed), the lack of studies to answer the research study is not at risk (i.e., 1914, cf., Table A1). This preliminary number is even expected to be enlarged along the searches on Lilacs and Scielo that are more ibero-American oriented. These results are expected to enable comprehensively mapping the evidence and regulatory elements of PCP on Portuguese healthcare services. At this level, methodological aspects of the individual studies included in the review might limit interpretation of their findings toward reliable identification of domains for improvement. Yet, in light of the preliminary number of retrieved records on MEDLINE, the study’s findings should at least enable pinpointing areas demanding further research to understand if PCP elements are being covered or not toward accomplishing PCP in Portuguese healthcare services. Accordingly, the results from the scoping review are expected to inform healthcare staff, educators, researchers, leaders, and policymakers about the domains of person-centeredness at the macro, meso, and micro levels that demand development in order to advance the PCP in Portugal.

## 4. Conclusions

This scoping review will aim to examine the PCP in the Portuguese healthcare services following the JBI method for conducting scoping reviews.

At the general level, this scoping review is expected to contribute to mapping the knowledge on PCP in the various domains of theories, care models, and regulation contributing to PCP in Portuguese healthcare settings. The comprehensive findings will then be discussed to allow for the identification of potential domains of improvement.

In light of the international advancements in PCP and the positive impact of its implementation to promote sustainable healthcare systems and high care quality, this scoping review is ultimately expected to contribute to raising the Portuguese quality of care to the international gold standard.

## Appendix A

**Table A1:** Draft of search strategy to Medline (PubMed) ^1^.

Search No.	Query	Records Retrieved
#1	((((((((health personnel[MeSH Terms]) OR (“health professional”[Title/Abstract])) OR (“healthcare professional”[Title/Abstract])) OR (physician[Title/Abstract])) OR (nurse[Title/Abstract])) OR (physiotherapist[Title/Abstract])) OR (nutritionist[Title/Abstract])) OR (psychologist[Title/Abstract])) OR (midwife[Title/Abstract])	827,852
#2	(((((((Humans[MeSH Terms]) OR (Patients[MeSH Terms])) OR (Persons[MeSH Terms])) OR (relative[Title/Abstract])) OR (family[Title/Abstract])) OR (individuals[Title/Abstract])) OR (client[Title/Abstract])) OR (subject[Title/Abstract])	21,125,847
#3	((((((“Patient-Centered Care”[Mesh Terms]) OR “Delivery of Health Care”[Mesh Terms]) OR (family-centered[Title/Abstract])) OR (people-centered[Title/Abstract])) OR (person-centered[Title/Abstract])) OR (client-centered[Title/Abstract])) OR (citizen-centered[Title/Abstract])	1,182,353
#4	((((Health services*[MeSH Terms]) OR (“health institution”[Title/Abstract]) OR (“healthcare institution”[Title/Abstract]))) OR ((“Primary Health Care”[Mesh]) OR (“Ambulatory Care Facilities”[Mesh])) OR ((“Assisted Living Facilities”[Mesh]) OR (nursing homes[Title/Abstract])) OR (hospital*[Title/Abstract]))	5,686,278
#5	((“Portugal”[MeSH Terms]) OR (“Portugal”[All Fields])) OR (“Portuguese” [All Fields]) OR (Portug*[All Fields])	239,152
#6	#1 AND #2 AND #3 AND #4 AND #5	1914

^1^ Search date: 7 December 2021.

## Appendix B

**Table A2:** Draft of data extraction matrix.

Main Field	Extraction Categories	Category Description
Literature ID	1. Reference number	Please insert
	2. Authors	Please insert
	3. Year	Please insert
	4. Title	Please insert
	5. Type of reference	1. Primary research 2. Unpublished research 3. Policy 4. Standard 5. Evidence-based guidelines 6. Text/opinion paper
	6. Research studies ID	e.g., journal, issue no., vol. no
	7. Policy ID	
	8. Standard ID	e.g., entity, domain
	9. Guidelines ID	e.g., entity, practice, healthcare setting
	10. Text/opinion paper ID	e.g., expert domain
Inclusion/Exclusion criteria	11. Does the literature refer to a healthcare practice/intervention, or approach to provide healthcare?	1: Yes 2: No
	12. Does the healthcare practice occur in a Portuguese healthcare context?	1: Yes 2: No
	13. Does the literature involve healthcare professionals?	1: Yes 2: No
	14. Does the healthcare practice/intervention or approach to provide healthcare include person-centeredness elements at the structural, processual or outcomes levels?	1: Yes 2: No
	15. Are there any other reasons for exclusion?	1: Yes 2: No
	16. Inclusion of paper	1: Yes 2: No
Characteristics of population	17. Who are the healthcare professionals in the healthcare practice/intervention?	1. Physicians 2. Nurses 3. Psychologists 4. Physiotherapists 5. Psychologists 6. Nutritionists 7. Midwifes 8. Multidisciplinary team 9. Other, please specify
	18. Who are the healthcare service users involved in the healthcare practice/intervention?	1. Persons of all ages 2. Patients of all ages 3. Relatives 4. Family 5. Community groups 6. Residents 7. Other, please specify
Characteristics of PCP structure	19. What is the policy associated with the healthcare practice/intervention?	1. Please describe 2. No policy is identified
	20. What is the standard associated with the healthcare practice/intervention?	1. Please describe 2. No standard is identified
	21. What are person-centeredness elements at the organizational level?	1. Please describe 2. Not explicitly stated
	22. What are the person-centeredness elements in relation to the care environment?	1. Please describe according to the PCPF 2. Not explicitly stated
	23. What are the evidence-based guidelines associated with the healthcare practice/intervention?	1. Please describe 2. Not explicitly stated
Characteristics of PCP process	24. What is the conceptual or theoretical framework guiding the healthcare practice/intervention?	1. Please describe
	25. What are the person-centered processes associated with the healthcare practice/intervention?	1. Narrative 2. Therapeutic partnership 3. Health plan 4. Shared decision-making 5. Authentic engagement 6. Sympathetic presence 7. Working holistically 8. Other, please specify
Characteristics of PCP outcomes	26. What are the person-centered outcomes being assessed?	1. Effectiveness, please specify 3. Process, please specify 2. No outcomes are assessed
	27. What are the measurement instruments being used to assess the person-centered outcomes?	1. Please describe 2. No outcomes are assessed
Characteristics of context	28. What is the healthcare setting domain?	1. Primary care 2. Community care 3. Elderly care 4. Specialized care, please specify 5. Other, please specify.
Literature summary		
Reviewers commentaries		
